# Idiopathic Colonic Varices: Case Report and Review of Literature

**DOI:** 10.5812/hepatmon.18916

**Published:** 2014-07-02

**Authors:** Ion Dina, Carmen Fierbinteanu Braticevici

**Affiliations:** 1 Department of Gastroenterology, St. John Emergency Hospital, “Carol Davila” University of Medicine and Pharmacy Bucharest, Bucharest, Romania; 2 Department of Gastroenterology, Emergency University Hospital Bucharest, “Carol Davila” University of Medicine and Pharmacy Bucharest, Bucharest, Romania

**Keywords:** Colonic Varices, Portal Hypertension, Colonoscopy

## Abstract

**Introduction:**

Colonic varices represent a very rare entity, either an incidental finding at colonoscopy or discovered due to its complication, the lower gastrointestinal bleeding. The most common cause of colonic varices is portal hypertension associated with liver disease or secondary to pancreatic conditions, like chronic pancreatitis or malignancies. The incidence of colonic varices is very low, even in liver cirrhosis where the patients frequently develop varices in the upper gastrointestinal tract, but surprisingly uncommon present with varices localized in the colon.

**Case Presentation:**

We report a case of idiopathic colonic varices, diagnosed at a routine colonoscopy performed for nespecific abdominal disturbances in a female patient without liver disease or pancreatic conditions responsible for portal hypertension development.

**Conclusions:**

The development of colonic varices in the absence of a certain trigger represents a major issue for practitioners due to its major complication, lower gastrointestinal bleeding.

## 1. Introduction

Idiopathic colonic varices are defined by the presence of dilated submucosal veins in the colon, in the absence of an identifiable trigger and not related to other medical conditions (1). There are very few data regarding idiopathic colonic varices in the literature, with 30 cases reported by English authors (1). Idiopathic colonic varices should be differentiated by the colonic varices that occur secondary to portal hypertension either in liver cirrhosis or due to other conditions associated with portal vein obstruction (2-4). The most common cause of portal hypertension is of course, the chronic liver disease, namely hepatic cirrhosis of multiple etiologies –viral, toxic, metabolic and also its fatal complication, hepatocellular carcinoma. Besides these, several medical entities are associated with portal vein thrombosis and secondary development of portal hypertension. Hemodynamic factors, hypercoagulability states and thrombophilic disorders are responsible for the occurrence of thrombosis in different territories, including portal vein system (5). Myeloproliferative disorders, antiphospholipid syndrome, deficiency of protein C, S and antithrombin III, mutation of factor V Leyden are the most frequent cited causes of thrombophilic predisposition associated with portal vein thrombosis (5). Although multiple coagulability defects are reported in general population, portal vein thrombosis is not often encountered in non-cirrhotic patients (5). While the increase portal pressure is the most common cause responsible for varices development within the gastrointestinal tract, other causes less frequent of colonic varices are congestive heart failure, local or systemic sepsis, drugs, postsurgical states, pancreatitis or pancreatic cancer complicated with splenic vein thrombosis, distant malignancies or extrinsec compression of portal system (5, 6). When discussing about recto-colonic varices, it also should be take into account another distinct entity, the “so called” rectal or colonic varices secondary to idiopathic portal venous thrombosis or idiopathic portal hypertension (3, 7). The data concerning this condition is also scarce, with very few cases reported. The overall incidence of colonic varices, irrespective the etiology is around 0.07% (8). Colonic varices are diagnosed through colonoscopy performed routinely or in emergency for identifying the cause of a lower gastrointestinal bleeding (6, 9). If active bleeding is present during the colonoscopy, colonic varices may be misdiagnosed by confounding them with colonic polyps, carcinoma or even with a normal colonic appearance if varix are flattened by insufflation (6). This aspect is very important due to its serious consequences if biopsy is attempted. Regarding the etiology of idiopathic colonic varices, certain hypotheses were proposed: an inherited vascular anomaly, familial aggregation or venous malformations (4, 6). The gold standard of diagnosis for colonic varices is selective mesenteric angiography (10). This interventional technique is used for both diagnostic and therapeutic purposes. On one hand it allows to localize the colonic varices, to identify the source of the gastrointestinal hemorrhage if the bleeding is active and the bleeding rate exceeds 5 mL/min and on the other hand it offers certain therapeutic options like use of pharmacologic substances or embolic materials to stop the bleeding (6, 10).

## 2. Case Presentation

**Figure 1.**
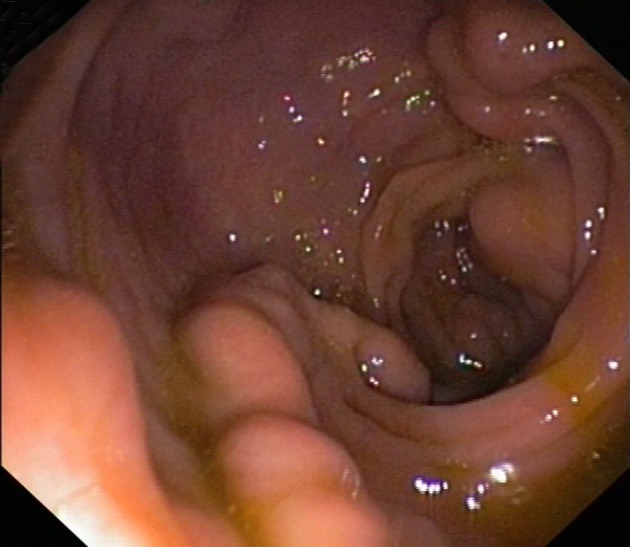
Extensive Colonic Varices.

**Figure 2.**
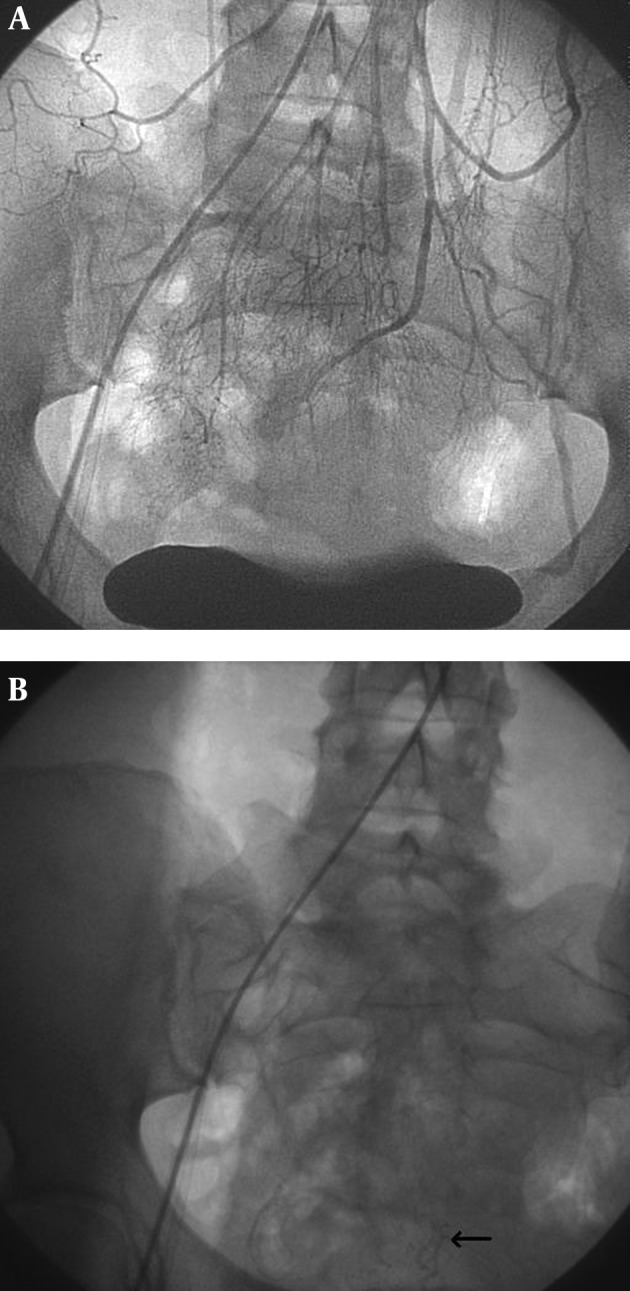
A) Angiography of inferior mesenteric artery: normal arterial phase. B) Angiography of inferior mesenteric artery: venous phase with blood stagnation suggestive for sigmoid varices.

A 38-year old female was admitted to our clinic for routine investigation of non-specific abdominal disturbances. The patient reported intermittent episodes of diarrhea without warning signs like passage of pus or blood into the stool, lasting for a couple of months. No frank rectal bleeding was mentioned. She denied abdominal pain, weight loss, fatigue or fever. Her personal history is unremarkable. Regarding the personal habits, she was non-smoker and no alcohol abuse or illicit drug use was noted. Family history was negative for colonic malignancies as well as for gastrointestinal bleeding. The physical examination revealed a well-looking pacient, not pale and normal weighted (BMI 26 kg/m^2^).The abdominal examination was unpainful, with no palpable masses, with normal liver and spleen. Laboratory investigations, including cell blood count and routine biochemical tests were within normal limits. Although no hepatic involvement was suspected after clinical examination, other serological markers for identifying liver diseases were ordered. The patient had no evidence for hepatitis B or C, for drug-induced liver disease or other specific liver diseases as hemochromatosis, alpha1-antitrypsin deficiency, Wilson’s disease, autoimmune liver disorders. Taking into account the digestive symptoms, an investigation of lower gastrointestinal tract was imposed. Colonoscopy was performed and showed dilated submucosal veins throught the entire colon, a finding consistent with extensive colonic varices (Figure 1). In order to clarify the etiology of colonic varix, supplementary investigations were undertaken. Abdominal ultrasound evidenced a normal appearance of the liver, spleen and pancreas, without other pathological findings; no signs of thrombosis of the portal axis were detected in Doppler examination. Abdominal Doppler sonography revealed normal portal flow volume and velocity; Acoustic radiation force impulse (ARFI) elastography showed that the mean value of liver measurements was 1.08 m/s (no liver fibrosis). Other blood analysis like clotting studies, autoantibody profile and imunoglobulins were in normal ranges. Upper digestive endoscopy did not reveal the presence of varices in the upper part of the gastrointestinal tract. A small bowel series was performed, but did not detect lacunar images suggestive for intestinal varices. In order to confirm the diagnosis, a selective angiography of inferior mesenteric artery was performed. The results exhibited a normal enhancement in arterial phase (Figure 2 A), while the venous phase showed stasis of the contrast medium within the descendent colon (Figure 2 B).

## 3. Discussion

Colonic varices describe the enlarged and dilated vessels localized in colonic submucosa that occur most frequent in association with portal hypertension secondary to significant liver disease (1, 11). Colonic varices should be considered “idiopathic” after other medical conditions either or not related to portal hypertension or portal vein thrombosis have been ruled out. The most common segments of varicosities development within the lower gastrointestinal tract are cecum and rectum, while in our patient-case, we deal with extensive, pancolonicvarices (12). Colonic varices may present with serious complications, intermitenthematochezia or severe rectal bleeding and diagnosed accordingly, or discovered incidentally during a colonoscopy performed for gastrointestinal tract symptoms, as in our female patient (3). Lower gastrointestinal bleeding from colonic varices is very rare with fewer than 100 cases reported in the literature (1, 3). Even in liver cirrhosis, bleeding from colonic varices appears an uncommon manifestation, while upper digestive hemorrhage through esophageal varix rupture is recognized as a frequent complication (4). In our patient case, there was no evidence of chronic liver disease, neither clinical nor biological and imagistical. There were no peripheral signs of liver cirrhosis, the liver presented with normal consistency at clinical examination and there where no abnormalities in hepatic function tests; in addition the serological markers for hepatitis B and C were negative. So, the most common cause of colonic varices, portal hypertension secondary to liver cirrhosis was excluded from the very beginning. Portal hypertension secondary to portal venous obstruction of different etiologies had been ruled out step by step. A thrombophilic state was excluded following specific biological investigations, coagulation analysis and autoantibody profile. The common cause of hypercoagulability encountered in young women population is contraceptive use, but this issue had not been taken into account because the patient did not mention this drug type administration. The other causes responsible for portal hypertension development (heart failure, pancreatic conditions, malignancies, sepsis, hematological diseases) were not supported by clinical, biological and imagistic data (5). In our case, the diagnosis of colonic varices was made be colonoscopy and confirmed by angiographic studies, which represents the most accurate diagnostic tool (10). The appearance of colonic varix in endoscopic examination was quite typical and the diagnosis was easily made. The differentiation of varix from other entities like polyps, carcinoma or ulcerative colitis was not an issue in this case. The only problem to solve was to establish the etiology of colonic varices. As we discussed above, we ruled out the most common cause, liver cirrhosis accompanied by portal hypertension, portal vein occlusion with secondary increase in portal pressure and other miscellanous causes. So, the final diagnosis was idiopathic colonic varices, which is a very rare condition as the majority of authors suggested. Due to the paucity of data, the treatment of colonic varices is not well defined (1, 4, 12). Idiopathic colonic varices occur more frequently in male patients, with a mean age at diagnosis about 41 years, pattern which is not respected in our case, outlining one more time the rarity of the case. As some authors stated, only 22 cases of idiopathic colonic varices were reported in patients without liver dysfunction, pancreatic conditions, abdominal surgery or congestive heart failure (6, 13, 14). A conservative approach is attempted in uncomplicated cases, while when presenting with major bleeding, partial colectomy is required (1, 8). In our case, we did not prescribe any treatment but we strongly recommend to present rapidly to the hospital in case of rectal bleeding.

The particularity of the case is the the rarity of the condition, the development of colonic varices in the absence of portal hypertension or portal vein thrombosis is considered an infrequent finding. The importance of a correct diagnosis derives from a serious complication associated with colonic varices, severe lower gastrointestinal bleeding that could endanger the patient’s life and represents a medical emergency.
